# “How I would like AI used for my imaging”: children and young persons’ perspectives

**DOI:** 10.1007/s00330-024-10839-9

**Published:** 2024-06-20

**Authors:** Lauren Lee, Raimat Korede Salami, Helena Martin, Lavanhya Shantharam, Kate Thomas, Emily Ashworth, Emma Allan, Ka-Wai Yung, Cato Pauling, Deirdre Leyden, Owen J. Arthurs, Susan Cheng Shelmerdine

**Affiliations:** 1 Young Persons Advisory Group (YPAG), Great Ormond Street Hospital for Children, London, WC1H 3JH UK; 2 Kings College Hospital NHS Foundation Trust, London, UK; 3 Guy’s and St Thomas’ NHS Foundation Trust, London, UK; 4 St George’s Hospital, Blackshaw Road, Tooting London, London, UK; 5 Royal Hospital for Children & Young People, Edinburgh, Scotland UK; 6 Department of Clinical Radiology, Great Ormond Street Hospital for Children, London, WC1H 3JH UK; 7 Wellcome/ EPSRC Centre for Interventional and Surgical Sciences, Charles Bell House, 43-45 Foley Street, London, W1W 7TY UK; 8 University College London, Gower Street, London, WC1E 6BT UK; 9 UCL Great Ormond Street Institute of Child Health, Great Ormond Street Hospital for Children, London, UK, WC1N 1EH UK; 10 NIHR Great Ormond Street Hospital Biomedical Research Centre, 30 Guilford Street, Bloomsbury, London, WC1N 1EH UK

**Keywords:** Surveys and questionnaires, Artificial intelligence, Child, Young adult, Attitude

## Abstract

**Objectives:**

Artificial intelligence (AI) tools are becoming more available in modern healthcare, particularly in radiology, although less attention has been paid to applications for children and young people. In the development of these, it is critical their views are heard.

**Materials and methods:**

A national, online survey was publicised to UK schools, universities and charity partners encouraging any child or young adult to participate. The survey was “live” for one year (June 2022 to 2023). Questions about views of AI in general, and in specific circumstances (e.g. bone fractures) were asked.

**Results:**

One hundred and seventy-one eligible responses were received, with a mean age of 19 years (6–23 years) with representation across all 4 UK nations. Most respondents agreed or strongly agreed they wanted to know the accuracy of an AI tool that was being used (122/171, 71.3%), that accuracy was more important than speed (113/171, 66.1%), and that AI should be used with human oversight (110/171, 64.3%). Many respondents (73/171, 42.7%) felt AI would be more accurate at finding problems on bone X-rays than humans, with almost all respondents who had sustained a missed fracture strongly agreeing with that sentiment (12/14, 85.7%).

**Conclusions:**

Children and young people in our survey had positive views regarding AI, and felt it should be integrated into modern healthcare, but expressed a preference for a “medical professional in the loop” and accuracy of findings over speed. Key themes regarding information on AI performance and governance were raised and should be considered prior to future AI implementation for paediatric healthcare.

**Clinical relevance statement:**

Artificial intelligence (AI) integration into clinical practice must consider all stakeholders, especially paediatric patients who have largely been ignored. Children and young people favour AI involvement with human oversight, seek assurances for safety, accuracy, and clear accountability in case of failures.

**Key Points:**

- *Paediatric patient’s needs and voices are often overlooked in AI tool design and deployment.*
- *Children and young people approved of AI, if paired with human oversight and reliability.*
- *Children and young people are stakeholders for developing and deploying AI tools in paediatrics.*

## Introduction

Recent advancements in technology have intensified the drive for digital tools that enhance patient outcomes, with artificial intelligence (AI) and machine learning (ML) at the forefront [1–3]. Multiple publications have highlighted the high performance of AI tools across different medical disciplines [4–7], often matching or enhancing the accuracy of skilled professionals. This has led to a surge in optimism from investors regarding AI’s role in future healthcare services, reflected by the amount of venture capital funding received in the field of digital health (projected to be $36 billion USD by 2025 [8]), and with radiology based applications leading the way (now comprising 85% of all FDA regulatory approved AI algorithms for commercial use [9]).

The radiology community (including those who work within paediatric radiology) are generally positive about the prospect of AI for enhanced patient care [10]; however, the availability and development of AI tools for paediatric imaging remains nascent. There are currently few commercial offerings specifically designed for children [11], although those for fracture detection, for example, may have the most emerging promise for widespread use in the near future [12, 13]. Whilst concerns exist amongst healthcare professionals regarding the performance and unintended consequences of such tools [14–16], it is vital that patient’s voices are heard when developing and designing AI tools for their care [17].

Although several articles have surveyed adult patient’s views on AI usage in radiology [18–20], few have sought the opinions of children and young adults [21–23]. Of those that have, none were specific to medical imaging, and all conducted as small group interviews with only a limited number of participants, which may not have reflected wider views. The current generation, being “digital natives”, could have distinct views on AI that may not mirror those of adult patients, and these would be critical to recognise particularly before any paediatric radiology-specific AI implementation work.

This study, therefore, aims to bridge this knowledge gap by conducting a national study to evaluate the thoughts of children and young adults on AI, particularly when applied to their own medical imaging, with a focus on fracture detection as the most likely disease they will have had direct experience of themselves.

## Methods and materials

Ethical approval was not required for this voluntary questionnaire of public opinions. Although respondents were given the option to share their contact details for future public engagement activities, this detail was not specifically required to participate in the survey.

### Questionnaire

The survey was based on a validated questionnaire developed by Ongena et al [24] regarding (adult) patient views on the implementation of AI in radiology. Their survey was developed by methodologists in collaboration with radiologists and 155 (adult) patients undergoing diagnostic imaging tests. Their survey consisted of six domains (proof of technology, procedural knowledge, competence, efficiency, personal interaction and accountability) as a framework for further questions, each answerable using a 5-point Likert scale.

Our survey used their validated survey as a starting point for discussion across three patient and public engagement meetings held between May and October 2021 by the ‘FRACTURE Study Patient and Public Involvement & Engagement (PPIE) Steering Committee’ [25]. This committee consisted of three parent representatives, four young person representatives, the institution’s PPIE Manager for research (DL) and the lead researcher for this study (SS). The parents and young people on this steering group were self-selected volunteers from two larger PPIE groups (called the ‘Great Ormond Street Hospital for Children London Young Persons’ Advisory Group’ for research (GOSH YPAG) and ‘Great Ormond Street Hospital for Children Parent and Carer Advisory Group for research) with an interest in digital technology [26].

A “child and young persons” friendly version of the survey was co-created with the FRACTURE Study PPIE Steering Committee, and approved based on the collective group opinions of what was considered important and understandable topics to raise. Care was taken to adapt the language to suit a child with a reading age of at least approximately 10 years old and above. An upper limit of age for a ‘young person’ was established by consensus as 23 years old. The final survey was tested amongst members of the steering committee to ensure user-friendliness.

To attract public attention and understanding of the aims of this work, an accompanying short animation [27] explaining what the survey was asking for and how to take part was also developed in consultation with the steering committee. The final survey was conducted in English and hosted on the Google Forms platform with the accompanying animation and instructions included on the front landing page.

The survey contained a total of 31 questions (ESM Supplementary Table 1), which comprised of 4 basic questions on demographic details (age, gender, ethnicity, location), 2 questions about computer and AI experience/knowledge, then 2 questions about the respondent’s fracture history. We used a 5-point Likert scale to score 21 subsequent questions regarding preferences for use of AI in children’s imaging (12 questions were specific to the use of AI for musculoskeletal imaging and 9 relating to AI for cardiac, oncological and neurological imaging collectively), with another 2 open-ended responses for elaboration on opinions. Questions were categorised according to five themes, namely accuracy, responsibility, ethical issues, resource allocation, and collaboration.

### Dissemination

The survey was ‘live’ for a total period of 12 months, from 1 June 2022 to 31st May 2023, and intended for children and young persons up to 23 years of age. A link to the survey (including an attachment of a poster of the survey with a QR code for the link) was disseminated through various local and national contacts, including

- Emailing 200 UK primary and secondary school administrators (using publicly available email addresses posted on school websites, ensuring dissemination across the four UK nations and boroughs)
- Emailing Student union representatives for 100 UK universities
- Emailing YPAG (Young Person’s Advisory Group for Research) Generation R representatives [26]
- Hosting the link on the FRACTURE Study website [25] and Twitter feed
- Hosting the link on the Great Ormond Street Hospital website [28] and Twitter feed
- Hosting the link on the Brittle Bone Society website [29] and Twitter feed
- Word of mouth and ‘retweeting’ of the survey link via local contacts within the GOSH YPAG group, colleagues and study collaborators within radiology, orthopaedic and paediatric departments with children and access to local parental and children groups.

Two email reminders were sent to local and national contacts during the study period to encourage uptake and response rates to the survey.

### Data Analysis

Simple descriptive statistics were used (e.g., frequency, proportion of respondents etc.) and analysed in Excel for Microsoft Office to depict the frequency of responses. Free text comments, where appropriate, are included with summary descriptives in order to lend an understanding of children and young persons voices for each thematic topic.

## Results

### Respondent demographics

**Fig. 1:**
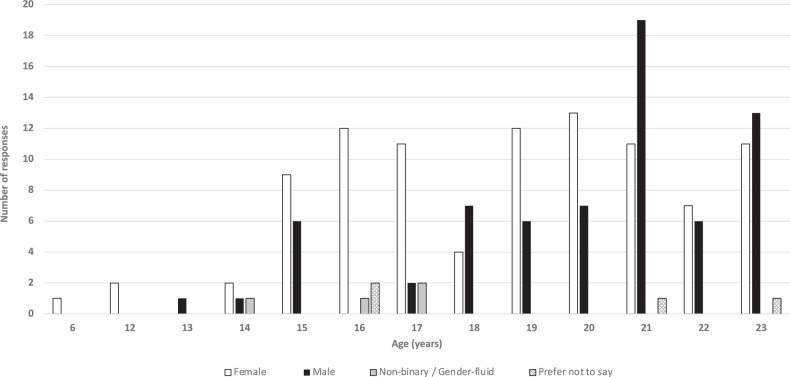
Bar chart demonstrating the spread of ages and genders of respondents to our survey (*n* = 171)

During the study period a total of 185 responses were received. 14 were excluded due to respondents being outside of the targeted age group leaving a total of 171 responses for analysis. Of these, 68 (39.8%) were male, and the average age of respondent was 19 years old (range 6–23 years of age) (Fig. 1).

**Fig. 2:**
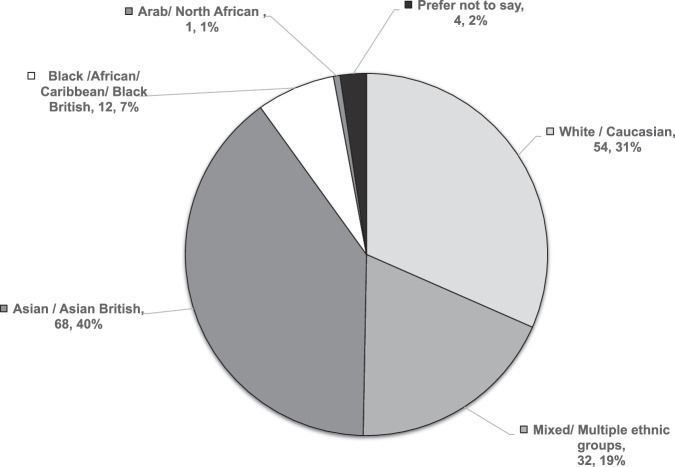
Pie chart depicting the self-reported ethnicity of the respondents (n, %) in this survey (*n* = 171)

We attracted respondents from a wide range of ethnicities, with the largest group of respondents being Asian/ Asian British (68/171, 39.8%) and White/ Caucasian (54/171, 31.6%) (Fig. 2). Responses were received from all four nations across the UK, representing 48/108 (44.4%) counties, with the majority located in London (27/171, 15.8%) and Cambridgeshire (15/171, 8.8%) (ESM Supplementary Table 2).

### Computer and AI literacy

The most frequent response given by respondents was that their computer skills were average (60/171, 35.1%), with only a few (12/171, 7%) stating they were advanced in their computing knowledge. When asked about their understanding of artificial intelligence in general, the most frequent response was that their understanding was average (58/171, 33.9%) or just below average (58/171, 33.9%), with again only a few (12/171, 7%) admitting they had a lot of knowledge in this area.

### Opinions on AI for medical imaging

**Fig. 3:**
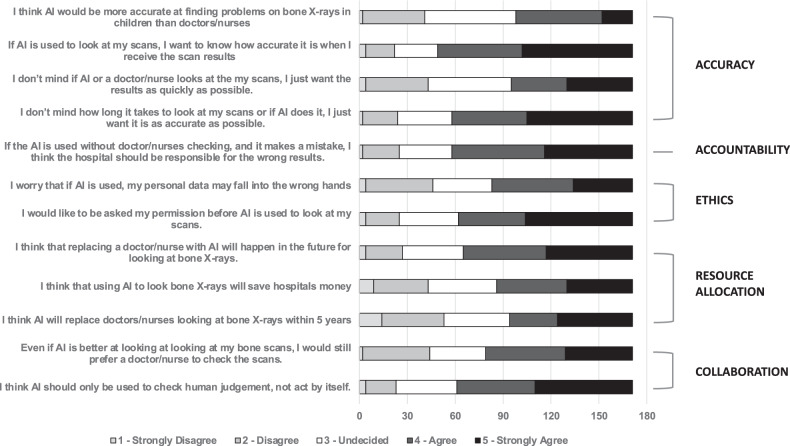
Bar chart demonstrating the opinions of respondents to the different questions relating to the use of AI for chlidren’s imaging in this survey (with particular relevance to “bone X-rays”). The proportions of respondents who either agreed or strongly agreed are down in darker colours versus those that disagree or strongly disagreed in lighter colours. Respondents who were undecided are depicted in white. Five key themes relating to the survey questions are shown on the right of the image

The full breakdown of all responses from the survey are outlined in Tables 1–3 and Fig. 3.

**Table 1:** FRACTURE survey summarised total responses (*n* = 171)

Theme / Survey Question	Likert Scale (1 = strongly disagree; 5 = strongly agree)						Average score
	1	2	3	4	5	No response	
**ACCURACY**							
I think AI would be more accurate at finding problems on bone X-rays in children than doctors/nurses	2 (1.2%)	39 (22.8%)	57 (33.3%)	54 (31.6%)	19 (11.1%)	0	3.29
**ACCURACY**							
If AI is used to look at my scans, I want to know how accurate it is when I receive the scan results	4 (2.3%)	18 (10.5%)	27 (15.8%)	53 (31.0%)	69 (40.4%)	0	3.96
**ACCURACY**							
I don’t mind if AI or a doctor/nurse looks at the my scans, I just want the results as quickly as possible	4 (2.3%)	39 (22.8%)	52 (30.4%)	35 (20.5%)	41 (24.0%)	0	3.89
**ACCURACY**							
I don’t mind how long it takes to look at my scans or if AI does it, I just want it is as accurate as possible	2 (1.2%)	22 (12.9%)	34 (19.9%)	47 (27.5%)	66 (38.6%)	0	3.41
**ACCOUNTABILITY**							
If the AI is used without doctor/nurses checking, and it makes a mistake, I think the hospital should be responsible for the wrong results	2 (1.2%)	23 (13.5%)	33 (19.3%)	58 (33.9%)	55 (32.2%)	0	3.82
**ETHICS**							
I worry that if AI is used, my personal data may fall into the wrong hands	4 (2.3%)	42 (24.6%)	37 (21.6%)	51 (29.8%)	37 (21.6%)	0	3.44
**ETHICS**							
I would like to be asked my permission before AI is used to look at my scans	4 (2.3%)	21 (12.3%)	37 (21.6%)	42 (24.6%)	67 (39.2%)	0	3.86
**RESOURCE ALLOCATION**							
I think that replacing a doctor/nurse with AI will happen in the future for looking at bone X-rays	4 (2.3%)	23 (13.5%)	38 (22.2%)	52 (30.4%)	54 (31.6%)	0	3.75
**RESOURCE ALLOCATION**							
I think that using AI to look bone X-rays will save hospitals money	9 (5.3%)	34 (19.9%)	43 (25.1%)	44 (25.7%)	41 (24.0%)	0	3.43
**RESOURCE ALLOCATION**							
I think AI will replace doctors/nurses looking at bone X-rays within 5 years	14 (8.2%)	39 (22.8%)	41 (24.0%)	30 (17.5%)	47 (27.5%)	0	3.33
**COLLABORATION**							
Even if AI is better at looking at looking at my bone scans, I would still prefer a doctor/nurse to check the scans	2 (1.2%)	42 (24.6%)	35 (20.5%)	50 (29.2%)	42 (24.6%)	0	3.51
**COLLABORATION**							
I think AI should only be used to check human judgement, not act by itself	4 (2.3%)	19 (11.1%)	38 (22.2%)	49 (28.7%)	61 (35.7%)	0	3.81

In this table, the number of responses and percentages of replies to all AI-related questions with Likert scale answers are provided *n*, (%). The final column provides the average score among all respondents

**Table 2:** FRACTURE survey—free text comments

Theme	Positive	Neutral	Negative
ACCURACY	“although most have been picked up- two tibial fractures have been missed and only picked up later once healing evident so the AI may well have picked up these” “I think that the use of AI would be beneficial for checking x rays and ensuring that fractures are spotted as doctors/nurses may sometimes miss them due to human error.” “I think that if AI are proven to be almost 100% more accurate than humans it should be introduced.”	“For the questions about the accuracy of AI, I do not feel that I know enough about how it works to give an opinion either way about the level of accuracy compared to that of a doctor’s work.”	
RESPONSIBILITY		“I think that AI could be extremely helpful but it is vital that we make sure they do not replace humans and their jobs. And also we make sure they are only doing minor jobs so if they mess up then a patient’s life isn’t ruined or risked”	
ETHICAL ISSUES		“I think that although AI is a powerful tool that can be utilized to save resources and time in hospitals, many decisions are still based around moral questions and ethical judgements, which a computer should not be relied on to answer those questions.”	“I believe that using AI may cause treatment to become less accessible to certain groups, as it may be designed from biased data. This could further separate the care each individual receives based on race etc.” “I do not think that AI should be involved in actual decision making as there are many ethical aspects involved that doctors are trained to tackle, it would only be acceptable if the AI was advanced enough to recognise these issues as a human would.” “the difference between technology (AI) and doctors or nurses is the ability to empathise. Technology cannot reassure the patient to show whether the results shown by AI is the accurate results because technology can’t show emotion.”
RESOURCE ALLOCATION			“I just wanted to add that hospitals will not save money by using AI, mainly due to how it is very hard to make a piece of tech that is exceptionally accurate for a cheap price. Also hospitals will still keep doctors and nurses anyway, and so used the tech as a double-checker in reality.”
COLLABORATION	“I think AI should be used to scan for fractures. I think AI should flag scans that are potentially more complex to interpret for review by radiologists.” “I think that AI should be used alongside a nurse/doctor to avoid any mistakes which will give the patient confidence whilst saving time.” “AI with a human check on diagnosis after seems best to me” “If doctors do not see what the issue is, they should put it in AI then. I had a fracture missed by two doctors, then a specialist noticed it, the first doctors could have used AI after not being able to figure out the source of my pain.” “AI could be used to filter out the most clear cut diagnoses and to flag all scans which may require more interpretation (lower percentage certainty of algorithm in its conclusion) for review by doctor/nurse”	“I strongly disagree with the use of the word ‚’replace’ when referring to AI in relation to nurses/doctors. I would hope there would always be collaboration between the 2 and AI would never be left to make decisions for the patient’s course of treatment alone.” “I believe if the AI detects anything too out of the ordinary it may be beneficial for a doctor to view it as well.” “I think there should be a balance between the use of AI and the HCPs also double checking the scans due to their increased insight and knowledge of the individual patient - allows for more personalised care”	“I think I’d feel the same way about every disease/body part, that there should at least be some minor oversight and that I feel a bit dubious about diagnostic potential”
GENERAL COMMENTS	“I feel its a great strategy and very unique” “This is very promising” “Great”	“ feel that a lot of factors that my opinions are dependent on are not clearly specified such as the resolution of the AI or whether it is the use of currently available AI or AI that may become available in the future”	“I think I would prefer a human to look at my scans if they were checking for cancer because of how sensitive that topic is” “There is still the fear of technology which causes us to rely on a doctor/ nurse, although, they can also make mistakes”

Free text comments from children and young people regarding how AI should be used in hospitals for looking at children’s imaging, categorised into themes and the nature of the comments

**Table 3:** Survey responses on use of AI for other pathologies (apart from fractures)

Question	Likert Scale (1 = strongly disagree; 5 = strongly agree)					
	1	2	3	4	5	No response
**I think AI would be more accurate than doctors/nurses for finding:**						
Cancer on scans	4 (2.3%)	55 (32.2%)	52 (30.4%)	45 (26.3%)	15 (8.8%)	0
Brain diseases on scans	7 (4.1%)	16 (9.4%)	47 (27.5%)	53 (31.0%)	47 (27.5%)	0
Heart diseases on scans	7 (4.1%)	25 (14.6%)	53 (31.0%)	50 (29.2%)	36 (21.1%)	0
**Even if AI is better at looking for cancer on my scans, I’d still prefer for a doctor/nurse to check the scans**.						
For cancer	7 (4.1%)	23 (13.5%)	21 (12.3%)	46 (26.9%)	74 (43.3%)	0
For brain diseases	6 (3.5%)	25 (14.6%)	31 (18.1%)	53 (31.0%)	56 (32.7%)	0
For heart diseases	8 (4.7%)	25 (14.6%)	32 (18.7%)	50 (29.2%)	56 (32.7%)	0
**I would be more willing to have AI look at my scans if they were checking for another disease (than bone problems)**						
For cancer	10 (5.8%)	35 (20.5%)	59 (34.5%)	36 (21.1%)	31 (18.1%)	0
For brain diseases	10 (5.8%)	27 (15.8%)	57 (33.3%)	40 (23.4%)	37 (21.6%)	0
For heart diseases	13 (7.6%)	28 (16.4%)	58 (33.9%)	31 (18.1%)	41 (24.0%)	0

In this table, the number of responses and percentages of replies to all AI-related questions with Likert scale answers are provided *n*, (%)

#### Accuracy

**Fig. 4:**
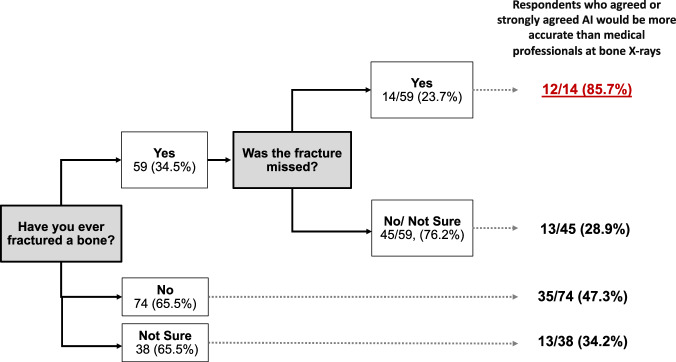
Flow chart demonstrating the differences in the proportion of respondents’ opinions regarding AI accuracy for bone X-rays relative to their own personal experience of sustaining a fracture

The most frequent response from respondents was that AI would be more accurate at finding problems on bone X-rays than medical professionals (42.7% (73/171) agree or strongly agree). In this survey 34.5% (59/171) of respondents said they previously had broken a bone, and of those, 14/59 (23.7%) reported that their fracture had been initially missed on X-ray. This subgroup were more likely than other respondents to think that AI would be more accurate than medical professionals at reviewing bone X-rays (Fig. 4).

The majority of respondents wanted to know how accurate the AI was when receiving their results (if AI was being used) (71.3% (122/171) agree or strongly agree) and accuracy was rated as more important than speed of results. In 66.1% (113/171), the respondents either agreed or strongly agreed that they would not mind how long it took to get results as long as they were as accurate as possible; whereas 44% (76/171) either agreed or strongly agreed with the statement that they wanted their results as quickly as possible (regardless of who assessed the examination).

Some free text comments relating to this domain included:

*“two tibial fractures have been missed and only picked up later once healing was evident so the AI may well have picked up these”*

*“I think that the use of AI would be beneficial for checking x rays and ensuring that fractures are spotted as doctors/nurses may sometimes miss them due to human error.”*

*“I think that if AI are proven to be almost 100% more accurate than humans it should be introduced.”*

#### Accountability

In general, most respondents believed that if the AI was used autonomously, and an error was made, then the hospital should be responsible for any inaccurate results (55/171, 32.2% strongly agreed, 58/171, 33.9% agreed). Nevertheless, the majority of respondents did not believe AI should act autonomously (see collaboration subheading below).

Some free text comments relating to this domain included:

*“I think that AI could be extremely helpful but it is vital that we make sure they do not replace humans and their jobs. And also we make sure they are only doing minor jobs so if they mess up then a patient’s life isn’t ruined or risked”*

#### Ethical issues

Most respondents (67/171, 39.2%) at this stage strongly agreed that they should be asked permission before AI was used on their imaging examinations, and there was still some mistrust amongst children and young adults about ‘personal data falling into the wrong hands’ as evidenced by 88/171 (51.5%) of respondents either agreeing or strongly agreeing with this statement.

Some free text examples were:

*“I think that although AI is a powerful tool that can be utilized to save resources and time in hospitals, many decisions are still based around moral questions and ethical judgements, which a computer should not be relied on to answer those questions.”*

*“I do not think that AI should be involved in actual decision making as there are many ethical aspects involved that doctors are trained to tackle, it would only be acceptable if the AI was advanced enough to recognise these issues as a human would.”*

*“the difference between technology (AI) and doctors or nurses is the ability to empathise. Technology cannot reassure the patient to show whether the results shown by AI is the accurate results because technology can’t show emotion.”*

#### Resource allocation

Many respondents either agreed (44/171, 25.7%) or strongly agreed (41/171, 24.0%) that using AI to look at bone X-rays would save hospitals money and that replacing a doctor or nurse with AI would happen in the future for this purpose (52/171, 30.4% agreed; 54/171, 31.6% strongly agreed). Most respondents thought this was likely to occur in the next 5 years (77/171 (45.0%) agreed or strongly agreed versus 53/171 (30.9%) disagreed or strongly disagreed).

*Despite the summary statistics above, a counter viewpoint was raised in free text comments:*

*“… hospitals will not save money by using AI, mainly due to how it is very hard to make a piece of tech that is exceptionally accurate for a cheap price. … hospitals will still keep doctors and nurses anyway, and so use the tech as a double-checker in reality.”*

#### Human/machine collaboration

Most respondents felt that AI should only be used to check human judgement but not act autonomously (61/171, 35.7% strongly agreed; 49/171, 28.7% agreed) and the majority of respondents either agreed (50/171, 29.2%) or strongly agreed (42/171, 24.6%) with the statement that ‘even if AI was better at looking at bone imaging, they would still prefer a healthcare professional to check the scans’.

Some positive comments were:

*“If doctors do not see what the issue is, they should put it in AI then. I had a fracture missed by two doctors, then a specialist noticed it, the first doctors could have used AI after not being able to figure out the source of my pain.”*

*“AI could be used to filter out the most clear cut diagnoses and to flag all scans which may require more interpretation (lower percentage certainty of algorithm in its conclusion) for review by doctor/nurse”*

Some neutral comments were:

*“I strongly disagree with the use of the word ‚’replace’ when referring to AI in relation to nurses/doctors. I would hope there would always be collaboration between the 2 and AI would never be left to make decisions for the patient’s course of treatment alone.”*

*“I believe if the AI detects anything too out of the ordinary it may be beneficial for a doctor to view it as well.”*

### Uses of AI in imaging

When asked whether respondents would have similar feelings about using AI to diagnose diseases on imaging tests if the disease was relating to cancer, heart or brain diseases—most respondents strongly agreed that they would prefer human oversight but did not indicate strong opinions about whether they were more willing for AI to be used for any particular disease processes.

**Fig. 5:**
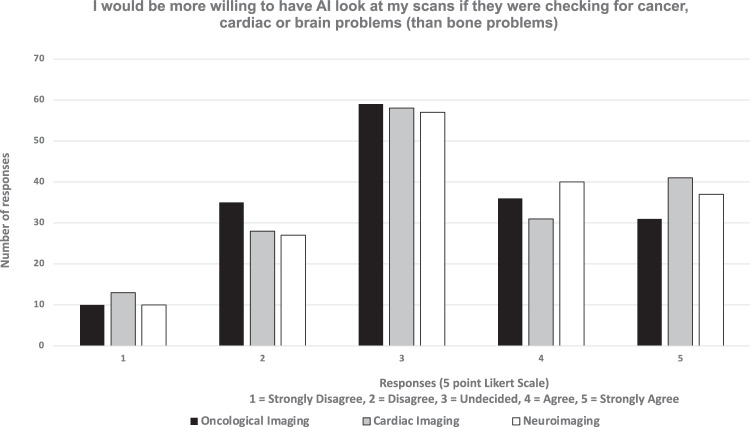
Bar chart showing the differences in opinions amongst the respondents about their willingness for AI to be used to interpret imaging for other diseases, such as cancer, cardiac or brain diseases. There did not appear to be a large difference in those agreeing or disagreeing for AI usage for the different diseases

**Fig. 6:**
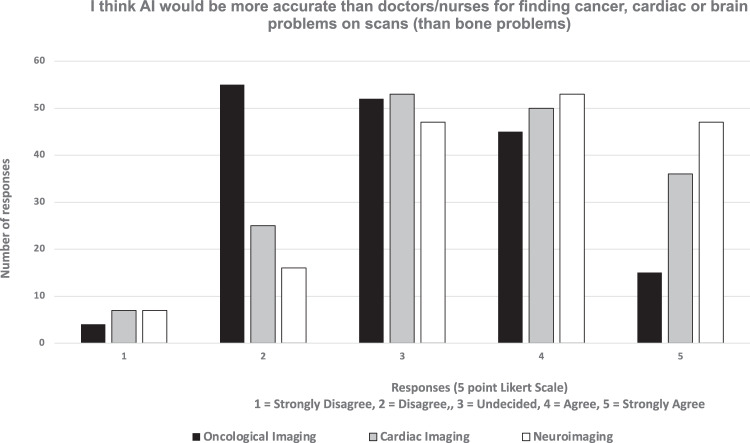
Bar chart showing the differences in opinions amongst the respondents about the perception of AI accuracy for detecting other diseases such as cancer, cardiac or brain diseases. Most respondents felt that AI was less likely to be accurate at identifying cancer than brain or heart diseases than healthcare professionals

Whilst most respondents felt that AI might be more accurate than humans at detecting brain diseases on scans (53/171, 31% agreed), they did not have strong opinions about whether or not AI would be more accurate at detecting cancer or heart diseases (52/171, 30.4%, and 53/171, 31%, respectively were undecided). See Table 3 and Figs. 5 and 6.

## Discussion

In this study, we found that children and young people were generally supportive of integrating AI into their healthcare journey, provided certain conditions were met. They expressed a strong interest in being informed about the accuracy of the AI tools being used, and conveyed a desire to be asked for consent for the application of AI on their medical images. Most children and young people expressed a preference for human oversight of the AI tool, though their willingness to accept AI assistance did not seem to vary greatly between different diseases as long as the outcomes were accurate. The respondents in this survey considered the accuracy of any imaging results to be more critical than the speed at which they were provided, in other words, respondents did not want accuracy to be compromised at the cost of speed.

Interestingly, respondents who had fractured a bone that was originally missed, were more likely to agree that AI could be more accurate than healthcare professionals at looking for bone problems on radiographs; whereas those who had fractured a bone (detected accurately) were the least likely to think that AI would be more accurate, presumably because they did not experience any particular delay or issue with their own diagnosis. This important finding highlights the need to ensure an appropriate mix of patient stakeholder engagement in any AI discussion, as personal medical and direct life experiences will affect perceptions about the potential for using an AI tool. We do acknowledge that the proportion of respondents in this study stating they had a ‘missed fracture’ may be higher than the general population (23.7% in this survey, versus an estimated 5–19% missed paediatric fractures by emergency clinicians (of which 5–28% are estimated to be clinically relevant) [30–34]. Emergency clinicians are usually the first healthcare professional to assess and discharge the patient, many times prior to formal radiology input in the UK). This higher rate in our survey could be for several reasons—those with missed fractures may have been more motivated to take part and many respondents found the link to our online survey via the ‘Brittle Bone Charity’ website, which supports patients with osteogenesis imperfecta. These patients have multiple fractures, many of which are missed and therefore their likelihood of a missed fracture may be higher than the average population. We know many children with this condition responded to our survey based on inference from free text comments, however due to preservation of respondent anonymity in our survey, we cannot quantify how many have this diagnosis.

There have only been a few publications exploring the opinions of children and young people on the use of AI for healthcare and none at present relating specifically to medical imaging. One study by Visram S et al [21] presented 21 members of a the wider GOSH YPAG with a variety of applications of AI in healthcare to understand areas they considered important for future adoption. Key themes surrounding governance, trust and human-centeredness were deemed important alongside patient empathy and safety. Another study by Thai K et al [22] interviewed 28 paediatric patients at a large urban children’s hospital and explored their opinions regarding the use of AI in clinical trials, clinical practice and health data research. A strong theme that emerged in this study was the need to maintain human interaction between patients and their physicians, although there were positive views relating to the use of AI for research and clinical care.

Whilst not AI-specific, there have been other surveys conducted on children’s views on the use of technology in general within healthcare [35], specifically for the use of robotics and virtual reality in hospital and educational settings [36–38]. In one scoping review looking at 73 publications relating to the use of robots in healthcare [37], it was found that the use of this technology was highly acceptable to children, parents and medical staff and feedback from robot usage was mostly positive. Although this type of technology differs from AI, it does support the acceptance that the younger generation have for interaction and integration of novel technologies for their own healthcare. In another review looking at 38 articles evaluating children’s concerns and needs in health technology (e.g. telehealth, medical devices, augmented reality) [39], four general overarching themes were found—issues relating to the stigma of using technology, data privacy, the trustworthiness of the technology and whether this was developed with age appropriateness in mind. Whilst the former may not be directly relevant for AI tools in imaging, the other three concerns do overlap with our survey findings where respondents expressed concern about the accuracy, security and trustworthiness of AI.

It is difficult to draw a direct comparison between adult and children’s views in the wider literature due to differences in questioning, nonetheless compared to the survey by Ongena et al [24] (upon which ours was based), adults on average more strongly agreed with the sentiment that it was important to get the scan results as fast as possible (score 4.49 in adults versus 3.89 for children (out of 5 on a Likert scale: 1 = strongly disagree, 3 = neutral, 5 = strongly agree)); scored similarly for worrying about data falling into the wrong hands (3.32 for adults versus 3.44 in children); and similarly for stating that even if AI was better at evaluating scans, they’d still prefer a doctor to review the study (3.32 for adults versus 3.51 in children). Regarding the fact that AI might replace doctors one day, adults scored an average of 3.50 versus 3.75 for children. The wish for faster results from the adult survey (compared to children) may reflect priorities in returning to work and life pressures (e.g. caring responsibilities). Other prior publications evaluating adult patient’s perceptions of AI in radiology have found similar thematic results to our survey of children and young adults. Most prefer human oversight of any AI tool and perceive any AI-based communication to lack emotional support, although they do welcome the use of AI if it can be proven to provide additional, accurate insights into their disease [24, 40, 41]. Other publications have additionally reported that a clear understanding of accountability and privacy concerns were a key factor in patient’s attitudes towards using AI-based healthcare solutions [18, 42, 43], including what using AI may mean with regard to clinical decision-making and access to healthcare professionals [44].

Comparing patient views (adults and children) with those of healthcare professionals on AI in imaging is challenging given the different focus of survey questions. Nonetheless, some similarities are noted—in one survey of healthcare professionals working in paediatric radiology [10], most agreed that their jobs were not at risk (85.4%), and that AI results should be still checked by a human (83.3% agreement). They also stated that diagnostic accuracy (32.1%), workflow efficiencies/speed (25.0%) and safety (22.5%) were the most important factors for consideration in AI design and implementation. In a different study of medical students [45], most (56%) were not convinced that AI could help with establishing definitive diagnoses in medicine and most agreed (83%) that AI would not replace radiologists. Finally, those working in mostly IT and industry were less trusting of AI, with only 25% stating they had confidence of AI results and 17% believing that the use of AI would mean healthcare staff could spend more time with patients, although they had high expectations of AI in the future with 86% believing medicine could become more efficient [46].

There are several limitations of our study. Our open recruitment strategy may have introduced a bias in the type of respondent that came forward to complete our survey. These are likely to have been from families and schools where access to digital devices and the internet were more accessible, those with English as a primary language and, potentially those from higher socioeconomic backgrounds (although we did not specifically ask about this detail). Our demographic representation may therefore not have included all possible ethnic backgrounds, although we received a large number of Asian participants, and our respondents did come from all four nations of the UK, indicating a broad reach of this survey.

We also focussed our survey mainly on opinions about AI for the diagnosis musculoskeletal disease on radiographs because we believed this was the most realistic clinical scenario for AI usage in the near future and a common disease many respondents would be familiar with. Whilst we did ask questions on more general areas, including cancer, brain and heart disease, we acknowledge that our findings may not generalise to all areas of paediatric imaging and more specific surveys on AI usage for those particular areas may be required. Furthermore, whilst we asked children to self-rate their computer literacy skills and awareness about AI, we do understand this is subjective, and there could be mixed perceptions about what ‘AI’ actually means. Nonetheless, from recent research conducted by a communications regulator in the UK (Ofcom) [47, 48], it was found that 59% of 7–17-year-old internet users have used a generative AI tool in the past year, with various international initiatives now promoting AI education programmes in school [49, 50], suggesting growing awareness and appreciation for this technology.

Our survey questions sought to strike a balance between being comprehensive, feasible but also understandable and not too tedious for children and young people to answer. To this end, we had to limit the number of questions we could ask, which we based these on the areas of priority guided by our GOSH PPIE Steering Committee. Future work could include smaller focus group or individual interviews with children and young people to delve deeper into some of the core issues surrounding accountability and ethical considerations to get more granular details on their opinions for these areas, in addition to exploring further their understanding of medical imaging tools and reasons for missed diagnoses when attending hospital. A survey exploring parental / carer viewpoints of the same questions may also be helpful in understanding if there is a difference between those of adults versus children, and whether there is further work needed to satisfy the needs of both (i.e. parent/carer and child) in the healthcare setting when considering AI implementation. Past studies reviewing caregiver and parental opinions on novel technologies in healthcare (e.g. robotics, virtual realtiy [38, 51, 52]) have generally shown high acceptance rates provided there are proven patient benefits with a careful consideration of possible risks of harm and how to mitigate these.

Finally, our survey was limited in our response rate due to limited uptake (although it still remains the largest survey of children and young persons views of AI for their medical imaging). Our survey, despite being based on an adult-validated survey of AI opinions for imaging and adapted with our PPIE steering group committee was not itself validated [53], and we had a wide range of ages of respondents with few non-adolescents. These included a single respondent aged 6 years old, two 12-year-old females, one 13-year-old male. Nevertheless, re-reviewing these individual concerns and replies to the survey questions did not deviate from those of the wider group.

In conclusion, children and young people in our survey population indicated that AI should be integrated into modern healthcare with an overwhelming preference for medical professional oversight for checks and balances. Our key messages from this survey should be considered by any hospital or radiology department looking to implement AI tools for children and young people so that their opinions and views are not forgotten. Further research into some aspects covered by our survey (e.g. ethical implications and accountability) from a wider population of respondents, or in depth subject-specific surveys, may be of benefit for future research.

## Supplementary information

Electronic Supplementary Material

## Abbreviations

AI: Artificial intelligence
FDA: Food and Drug Administration
GOSH YPAG: Great Ormond Street Hospital Young Persons’ Advisory Group for research
PPIE: Patient and public involvement and engagement
UK: United Kingdom
YPAG: Young Persons Advisory Group for research
