# Supplementary material for: “How I would like AI used for my imaging”: children and young persons’ perspectives
Source: Eur Radiol. 2024 Jun 20;34(12):7751–64. doi: 10.1007/s00330-024-10839-9 (PMC11557655; doi:10.1007/s00330-024-10839-9)
Supplement: Supplementary file 1 — Electronic Supplementary Material [file 330_2024_10839_MOESM1_ESM.pdf]

“How I Would like AI Used for my Imaging”: Children and Young Persons’ Perspectives

Electronic Supplementary Material (ESM)

Eur Radiol (2024) Lee L, Salami RK, Martin H et al.

Supplementary Table S1: FRACTURE Survey: Should AI be used to make decisions in hospitals?

QUESTION	RESPONSE ALLOWED
What is your gender?	Female / Male / Non-binary / Prefer not to say / Other
What is your age?	Open-Ended Response
What is your ethnicity?	White/Caucasian<br>Mixed/Multiple ethnic groups<br>Asia/ Asian British<br>Black/ African/ Caribbean/ Black British<br>Prefer not to say<br>Other
Where do you currently live (city, country)?	Open-Ended Response
How do you rate your computer skills?	Likert Scale 1 – 5; 1 = not very good, 5 = excellent
How much knowledge do you already have about artificial intelligence in general?	Likert Scale 1 – 5; 1 = nothing at all, 5 = a lot
Have you ever broken a bone before?	Yes/ No
Was the broken bone initially missed on X-ray tests?	Yes/ No/ Not Sure/ I have not broken a bone before (not applicable)
I think AI would be more accurate at finding problems on bone X-rays in children than doctors/nurses.	Likert Scale 1 – 5; 1 = strongly disagree, 5 = strongly agree
Even if AI is better at looking at looking at my bone scans, I would still prefer a doctor/nurse to check the scans.	Likert Scale 1 – 5; 1 = strongly disagree, 5 = strongly agree

I think AI should only be used to check human judgement, not act by itself.	Likert Scale 1 – 5; 1 = strongly disagree, 5 = strongly agree
I worry that if AI is used, my personal data may fall into the wrong hands	Likert Scale 1 – 5; 1 = strongly disagree, 5 = strongly agree
If the AI is used without doctor/nurses checking, and it makes a mistake, I think the hospital should be responsible for the wrong results.	Likert Scale 1 – 5; 1 = strongly disagree, 5 = strongly agree
I would like to be asked my permission before AI is used to look at my scans.	Likert Scale 1 – 5; 1 = strongly disagree, 5 = strongly agree
If AI is used to look at my scans, I want to know how accurate it is when I receive the scan results	Likert Scale 1 – 5; 1 = strongly disagree, 5 = strongly agree
I don't mind if AI or a doctor/nurse looks at the my scans, I just want the results as quickly as possible.	Likert Scale 1 – 5; 1 = strongly disagree, 5 = strongly agree
I don't mind how long it takes to look at my scans or if AI does it, I just want it is as accurate as possible.	Likert Scale 1 – 5; 1 = strongly disagree, 5 = strongly agree
I think that replacing a doctor/nurse with AI will happen in the future for looking at bone X-rays.	Likert Scale 1 – 5; 1 = strongly disagree, 5 = strongly agree
I think that replacing a doctor/nurse with AI will happen in the future for looking at bone X-rays.	Likert Scale 1 – 5; 1 = strongly disagree, 5 = strongly agree
I think that using AI to look bone X-rays will save hospitals money	Likert Scale 1 – 5; 1 = strongly disagree, 5 = strongly agree
I think AI will replace doctors/nurses looking at bone X-rays within 5 years	Likert Scale 1 – 5; 1 = strongly disagree, 5 = strongly agree

Are there any extra comments or opinions you wish to make? For example, were there certain views that you feel particularly strongly about that you wish to explain in detail here?	Open-Ended Response
1. I think AI would be more accurate than doctors/nurses for finding cancer on scans	Likert Scale 1 – 5; 1 = strongly disagree, 5 = strongly agree
2. Even if AI is better at looking for cancer on my scans, I'd still prefer for a doctor/nurse to check the scans.	Likert Scale 1 – 5; 1 = strongly disagree, 5 = strongly agree
3. I would be more willing to have AI look at my scans if they were checking for cancer (than bone problems)	Likert Scale 1 – 5; 1 = strongly disagree, 5 = strongly agree
1. I think AI would be more accurate than doctors/nurses for finding brain diseases on scans	Likert Scale 1 – 5; 1 = strongly disagree, 5 = strongly agree
2. Even if AI is better at looking for brain diseases on my scans, I'd still prefer for a doctor/nurse to check the scans.	Likert Scale 1 – 5; 1 = strongly disagree, 5 = strongly agree
3. I would be more willing to have AI look at my scans if they were checking for cancer (than bone problems)	Likert Scale 1 – 5; 1 = strongly disagree, 5 = strongly agree
1. I think AI would be more accurate than doctors/nurses for finding heart diseases on scans	Likert Scale 1 – 5; 1 = strongly disagree, 5 = strongly agree
2. Even if AI is better at looking for heart diseases on my scans, I'd still prefer for a doctor/nurse to check the scans.	Likert Scale 1 – 5; 1 = strongly disagree, 5 = strongly agree

3. I would be more willing to have AI look at my scans if they were checking for heart diseases (than bone problems)	Likert Scale 1 – 5; 1 = strongly disagree, 5 = strongly agree
Are there any comments or opinions you wish to make? For example, were there certain views that you feel particularly strongly about that you wish to explain in detail here?	Open-Ended Response

Supplementary Table S2: Location of survey respondents (descending order of frequency)

County/Region:	n (%)	County/Region:	n (%)
UK, region not specified	6 (3.5%)	Isle of Man	5 (2.9%)
**England**			
England, region not specified	3 (1.8%)	Lincolnshire	2 (1.2%)
Avon	10 (5.8%)	London	28 (16.4%)
Buckinghamshire	3 (1.8%)	Merseyside	1 (0.6%)
Cambridgeshire	15 (8.8%)	Norfolk	1 (0.6%)
Cheshire	2 (1.2%)	North Yorkshire	3 (1.8%)
Cornwall	1 (0.6%)	Nottinghamshire	1 (0.6%)
Derbyshire	3 (1.8%)	Oxfordshire	5 (2.9%)
Devon	3 (1.8%)	Shropshire	1 (0.6%)
Durham	2 (1.2%)	Somerset	2 (1.2%)
East Sussex	2 (1.2%)	South Yorkshire	3 (1.8%)
Essex	2 (1.2%)	Staffordshire	3 (1.8%)
Hampshire	6 (3.5%)	Surrey	8 (4.7%)
Herefordshire	4 (2.3%)	Tyne and Wear	2 (1.2%)
Hertfordshire	2 (1.2%)	West Midlands	5 (2.9%)
Kent	2 (1.2%)	West Sussex	1 (0.6%)
Lancashrie	4 (2.3%)	West Yorkshire	3 (1.8%)
Leicestershire	1 (0.6%)	Wiltshire	2 (1.2%)
**Wales**			
Clwyd	1 (0.6%)	South Glamorgan	1 (0.6%)
Gwent	1 (0.6%)	West Glamorgan	1 (0.6%)
Gwynedd	2 (1.2%)		
**Scotland**			
Aberdeenshire	1 (0.6%)	Inverness-shire	1 (0.6%)
Angus	2 (1.2%)	Perthshire	1 (0.6%)
Edinburgh	2 (1.2%)	Stirlingshire	1 (0.6%)
Glasgow	1 (0.6%)		
**Ireland**			
City of Belfast	4 (2.3%)	County Down	5 (2.9%)
County Armagh	1 (0.6%)		
